# The Arsenic Differential: Metabolism Varies Between Children and Adults

**Published:** 2005-06

**Authors:** Tina Adler

Worldwide, millions of people drink water contaminated with arsenic, but not everyone who drinks contaminated water has the same severity of effects. It has long been speculated that this differing susceptibility to the adverse health effects of arsenic may be due to differences in the way people metabolize the element. Now researchers in Mexico and Arizona are finding that age may play a role as well: children may metabolize arsenic differently than adults, even if they share similar genetic traits **[*EHP* 113:775–781].**

When arsenic is metabolized, it takes on new chemical identities that vary widely in their toxicity. Enzymes attach a methyl group to arsenic and convert it first to monomethylarsenic and then to dimethylarsenic. During this process, arsenic also changes its valence (the configuration of its electrons), which can affect its toxicity. The way a person metabolizes arsenic is reflected in a pattern of relative concentrations of arsenic metabolites in urine.

In the current study, researchers investigated urinary arsenic patterns of healthy residents of the Yaqui Valley in Sonora, Mexico. Participants fell into two age groups: children aged 7–11 and adults aged 18–79. The arsenic concentrations in the participants’ water were between 5.5 and 43.3 parts per billion, a range found in many parts of the world. The researchers cataloged polymorphisms in a gene known to be involved in arsenic metabolism, arsenic (III) methyltransferase (*CYT19*), and used existing catalogs of polymorphisms in two others, purine nucleoside phosphorylase and glutathione-*S*-transferase omega. Then they tested the participants’ urine to see how a subset of 23 of these polymorphisms related to urinary arsenic levels.

Among the children, polymorphisms on *CYT19* were strongly associated with a particular pattern of metabolites: a high ratio of dimethylarsenic to monomethylarsenic. Indeed, children with three polymorphic sites on *CYT19* were much more likely than any other participants to have the high ratio. No statistically significant association was seen in the adults—children and adults could have the same polymorphisms on their *CYT19* gene, yet have different ratios of dimethylarsenic to monomethylarsenic in their urine. However, even children with nonvariant *CYT19* had a higher ratio of dimethylarsenic to monomethylarsenic than adults.

Finding the association between the polymorphisms and urinary arsenic patterns only in children indicates that the association may be developmentally regulated, the researchers suspect. The *CYT19* gene may turn on during a certain developmental stage and be more or less active at different ages in a way that depends on a person’s DNA sequence.

Whereas this study examined healthy subjects, planned follow-up studies will include individuals suffering from arsenic-related health effects to see if there is a relationship between their health effects, their urinary arsenic patterns, and their DNA sequences. The findings from such research may prove particularly important as new clinical uses for arsenic compounds are emerging in the area of cancer treatment, where differences in metabolism and toxicity are important to oncologists and their patients.

## Figures and Tables

**Figure:**
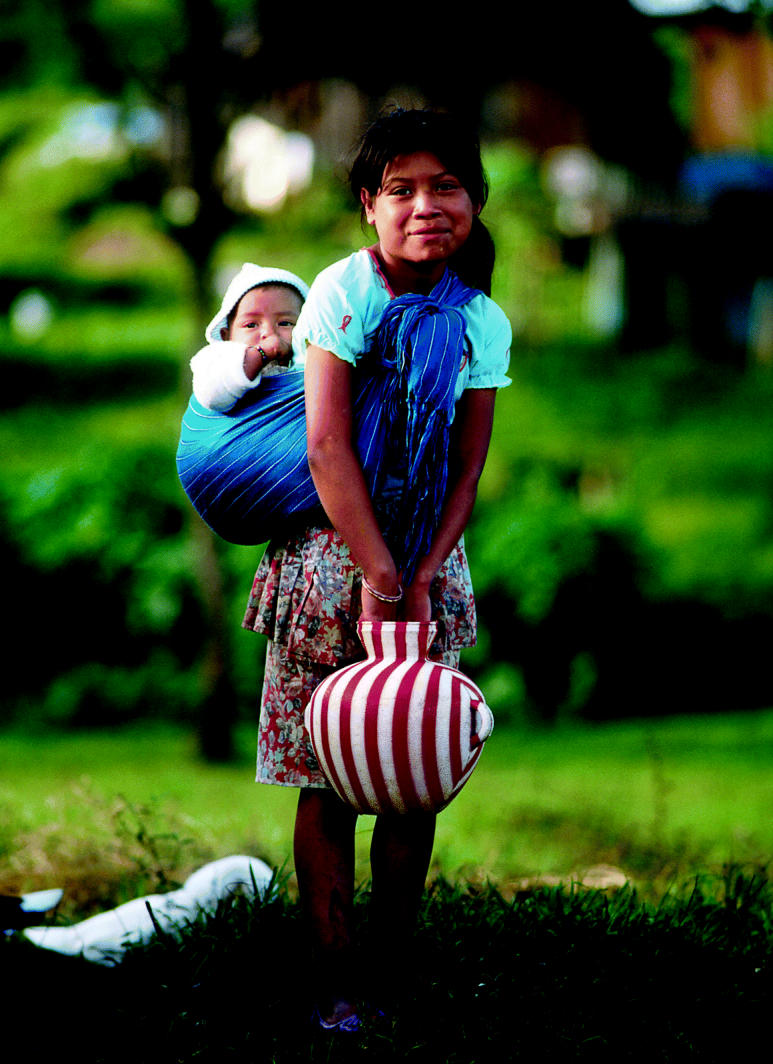
**New data on big and little dippers.** Research in Mexico reveals differences in the way adults and children metabolize arsenic from drinking water.

